# Using Vocal Characteristics To Classify Psychological Distress in Adult Helpline Callers: Retrospective Observational Study

**DOI:** 10.2196/42249

**Published:** 2022-12-19

**Authors:** Ravi Iyer, Maja Nedeljkovic, Denny Meyer

**Affiliations:** 1 Centre for Mental Health Swinburne University of Technology Hawthorn Australia

**Keywords:** machine learning, distress, voice, mental distress, psychological stress, artificial intelligence, emotional distress, voice biomarker, biomarker, digital health intervention, mental health, mental health intervention, psychological well being, speech analysis

## Abstract

**Background:**

Elevated psychological distress has demonstrated impacts on individuals’ health. Reliable and efficient ways to detect distress are key to early intervention. Artificial intelligence has the potential to detect states of emotional distress in an accurate, efficient, and timely manner.

**Objective:**

The aim of this study was to automatically classify short segments of speech obtained from callers to national suicide prevention helpline services according to high versus low psychological distress and using a range of vocal characteristics in combination with machine learning approaches.

**Methods:**

A total of 120 telephone call recordings were initially converted to 16-bit pulse code modulation format. Short variable-length segments of each call were rated on psychological distress using the distress thermometer by the responding counselor and a second team of psychologists (n=6) blinded to the initial ratings. Following this, 24 vocal characteristics were initially extracted from 40-ms speech frames nested within segments within calls. After highly correlated variables were eliminated, 19 remained. Of 19 vocal characteristics, 7 were identified and validated as predictors of psychological distress using a penalized generalized additive mixed effects regression model, accounting for nonlinearity, autocorrelation, and moderation by sex. Speech frames were then grouped using k-means clustering based on the selected vocal characteristics. Finally, component-wise gradient boosting incorporating these clusters was used to classify each speech frame according to high versus low psychological distress. Classification accuracy was confirmed via leave-one-caller-out cross-validation, ensuring that speech segments from individual callers were not used in both the training and test data.

**Results:**

The sample comprised 87 female and 33 male callers. From an initial pool of 19 characteristics, 7 vocal characteristics were identified. After grouping speech frames into 2 separate clusters (correlation with sex of caller, Cramer’s V =0.02), the component-wise gradient boosting algorithm successfully classified psychological distress to a high level of accuracy, with an area under the receiver operating characteristic curve of 97.39% (95% CI 96.20-98.45) and an area under the precision-recall curve of 97.52 (95% CI 95.71-99.12). Thus, 39,282 of 41,883 (93.39%) speech frames nested within 728 of 754 segments (96.6%) were classified as exhibiting low psychological distress, and 71455 of 75503 (94.64%) speech frames nested within 382 of 423 (90.3%) segments were classified as exhibiting high psychological distress. As the probability of high psychological distress increases, male callers spoke louder, with greater vowel articulation but with greater roughness (subharmonic depth). In contrast, female callers exhibited decreased vocal clarity (entropy), greater proportion of signal noise, higher frequencies, increased breathiness (spectral slope), and increased roughness of speech with increasing psychological distress. Individual caller random effects contributed 68% to risk reduction in the classification algorithm, followed by cluster configuration (23.4%), spectral slope (4.4%), and the 50th percentile frequency (4.2%).

**Conclusions:**

The high level of accuracy achieved suggests possibilities for real-time detection of psychological distress in helpline settings and has potential uses in pre-emptive triage and evaluations of counseling outcomes.

**Trial Registration:**

ANZCTR ACTRN12622000486729; https://www.anzctr.org.au/ACTRN12622000486729.aspx

## Introduction

In recent years, the presence of psychological distress in the community has escalated sharply due to COVID-19 [1,2]. Given the demonstrated impacts of high distress on health and functioning, reliable and efficient ways to detect distress are key to early intervention. Despite this growing problem, surprisingly little attention has been paid to methods for the detection of distress in the broader community. We aimed to use artificial intelligence to automatically classify a sample of helpline call recordings according to high and low psychological distress using voice characteristics alone. Thus, we offer an objective and efficient approach to the real-time detection of psychological distress in a large sample of helpline callers.

Psychological distress has been defined as “the unique discomforting emotional state experienced by an individual in response to a specific stressor or demand that results in harm, either temporary or permanent, to the person” [3]. Although commonly associated with diagnoses of depression and anxiety, psychological distress is also associated with other psychiatric diagnoses such as posttraumatic stress disorder (PTSD) and schizophrenia [4] and is a feature of significant life events such as bereavement and employment loss [5,6]. If left undetected, psychological distress can contribute to declines in physical and mental health, longer hospital stays, poor treatment compliance, and increasing cost of care [7].

Helplines internationally have witnessed substantial increases in call volumes and presentations involving psychological distress in recent years [8]. The support provided by helpline staff has been directly linked to sustained reductions in psychological distress [9]. However, the assessment of psychological distress is challenged by the absence of nonverbal cues, time limitations, and reticence of some callers to discuss relevant issues. Helpline staff have also reported difficulties when asking callers directly about psychological distress [10], suggesting a role for alternative approaches to the detection of psychological distress in helpline settings.

Pilot data support the use of voice characteristics to identify psychological distress in varied settings. Scherer and colleagues [11] investigated whether voice recordings could be separated between high and low psychological distress using vowel space as a measure of clarity of articulation and expressiveness of speech (hypothesizing that greater vowel space would signify psychomotor slowing of speech production due to psychological distress). In this study, participants were sourced from 2 separate databases. The first of these was the Distress Assessment Interview Corpus, which contains audio-visual recordings of semistructured interviews conducted between 253 participants and a virtual interviewer, coded for depression using the Patient Health Questionnaire-9, and PTSD using the PTSD-checklist civilian version. A second sample of 68 recordings was also analyzed, sourced from an audio-visual database of interviews with both participants with and without depression. Small to moderate effect sizes were found in comparisons of depressed and nondepressed recordings (Hedge’s G–0.43) and in those of PTSD with an absence of PTSD diagnosis (Hedge’s G=–0.34). This study indicated that psychologically distressed individuals may have poorer articulatory clarity and expressiveness and therefore suggested voice characteristics as a viable marker of psychological distress more broadly.

Kansberger and colleagues [12] alternatively hypothesized that the average vocal pitch would be elevated when utterances were compared between psychologically distressed and emotionally neutral conditions. Average fundamental frequency values (lowest discernible pitch) extracted from follow-up interviews of 16 patients with head and neck cancer were coded for the presence of overt and implied statements (n=89) of psychological distress. A mixed effects logistic regression analysis found that fundamental frequency values were indeed elevated in the presence of psychological distress (*P*<.05; 95% CI 1.82-24.31Hz). Furthermore, elevations in pitch accounted for ~70% of the variance in psychological distress outcomes, suggesting that the results obtained were robust against differences between individual participants.

However, these studies reveal a number of limitations. As Kansberger and colleagues [12] note, fundamental frequency (or for that matter, any single measure) is unlikely to be singly prognostic of psychological distress. As Franklin and colleagues [13] note in their review of assessment measures for suicide risk, research would benefit from the use of predictive algorithms capable of considering multiple predictors simultaneously: a research direction that may well also apply to the optimal identification of psychological distress. Furthermore, although Kansberger et al [12] accounted for differences in pitch, nested within individual participants, the authors did not account for nonlinearity or correlation between utterances, suggesting a missed opportunity for accurate modeling of changes in pitch over time. Conversely, while Scherer et al [11] analyzed a larger sample of recordings, these were obtained from recordings of interviews conducted with a virtual avatar and may lack generalizability to more ecologically valid settings.

Artificial intelligence has the potential to detect states of psychological distress in an accurate, efficient, and timely manner using multiple measures. Although there is initial evidence for the efficacy of such an approach [14], current evidence lacks application to real-world ecologies and real-time assessment, both of which are essential if these insights are to move beyond the laboratory. Thus, we aimed to use artificial intelligence approaches to automatically classify a large sample of telephone counseling calls made to Australian suicide prevention helpline services according to the level of psychological distress using a range of vocal characteristics. By classifying short segments of each call to a high level of accuracy, we aim to demonstrate a viable artificial intelligence support to existing helpline infrastructure that can be deployed in real time.

## Methods

Multimedia Appendix 1 illustrates the steps taken in both preprocessing and the final analysis of the vocal characteristics.

### Call Recordings

A total of 537 call recordings were initially sourced from On The Line, Australia (the suicide call-back service) and the Australian Federal Police, Canberra (000 emergency services response), as part of a broader study classifying low from imminent risk of suicide using voice characteristics [15]. On The Line recordings were chosen at random from between July 1, 2019, and June 30, 2021, stratified by suicide risk level and disclosed sex of caller. The Australian Federal Police recordings were purposely chosen over the same time period to reflect imminent risk of suicide necessitating emergency services response.

### Preprocessing of Calls

All calls were received as mono-channel 8 kHz, 32-bit float format. Each recording was initially transformed to 16-bit pulse code modulation format and normalized (zero mean) with pre-emphasis added to attenuate low signals and emphasize higher frequency signals, thus clarifying the degree of audibility, particularly where this was compromised (eg, mobile phone calls with considerable background noise). Listwise removal of missing data (silence; 381,655 of 722,274 segments, 52.84%) occurred prior to the subsequent analyses.

### Selection of Call Recordings Relating to Psychological Distress

A multigated approach informed the designation of psychological distress ratings to each annotated segment of each call recording. A team of associate researchers (n=6) assigned distress levels to segments selected from within each call as described in the next section. The associate researchers were either provisional or fully registered psychologists (henceforth referred to the “psychologists”) with the Psychology Board of Australia, completing postgraduate qualifications in psychology and who had substantial prior experience working with complex presentations, often involving psychological distress of varying magnitude (eg, psychiatric diagnoses, significant life events). A random sample of the 120 call recordings was assigned to each of the psychologists, with 20% crossover (4 of 22 calls assigned to each rater) used for the assessment of interrater reliability (Cohen *κ*=0.92).

The psychologists were asked to annotate segments of each recording using audio software (Audacity, version 2.4.2, The Audacity Team). Annotated segments were to be free from the counselor’s voice as much as possible and were to feature a diversity of psychological distress ratings; thus, it was common for a range of distress ratings to feature across annotated segments within each call recording, ensuring our analysis considered within-caller variation of distress. Psychological distress was rated by each associate researcher using the distress thermometer, an 11-point discrete Likert-style scale (0=no distress and 10=extreme distress). The distress thermometer has a critical level of 4 (SD 1.5) indicating clinical levels of psychological distress that require follow-up and referral [16]. Thus, the ratings of psychological distress for each segment were dichotomized for either side of the recommended clinical psychological distress rating (distress rating=4). The psychologists also described each caller’s presentation using standardized psychiatric mental status examination descriptions.

The distress thermometer has been used extensively in areas other than oncology, for which the scale was first developed, with good concordance achieved between clinician and patient ratings (n=364, Lin’s coefficient of concordance=0.79, sensitivity=0.83 and specificity=0.71) [17]. The distress thermometer has been compared with other well-validated measures for identifying psychological distress including the Hospital Anxiety and Depression Scale, the Structured Clinical Interview for DSM (Diagnostic and Statistical Manual of Mental Disorders) disorders and the Depression Anxiety and Stress Scale-21 [16], with a mean area under the receiver operating characteristic curve (AUROC) of 0.82 (SD 0.07) across all these measures [18].

### Power Analysis

Following Zou [19], the intraclass correlation coefficient from Kandsberger [12] (intraclass correlation coefficient=0.31) was used with a theoretical number of distress annotations per call (n=4) indicating a desired sample size of 119 calls necessary to identify a significant main effect for psychological distress (power=0.8, *α*=.05). Thus, 120 calls were chosen at random from the overall sample of callers.

### Derivation of Vocal Characteristics

Twenty-four unique vocal characteristics were initially derived from the digital vocal using the analyze function of the Soundgen package in RStudio (version 2022.07.01). Nineteen vocal characteristics remained after removal of highly correlated variables. Annotated segments of each recording were divided into overlapping (50%) 40-ms Blackman-windowed frames, with the segment level of psychological distress assigned to all frames within each segment. The choice of frame size provided a level of focus on important characteristics of the soundwave by magnifying central frequencies of each frame and ensuring that valuable information was not lost in the tails of each window.

### Selection and Validation of Candidate Vocal Characteristics

A penalized 2-level generalized additive mixed-effects regression model (GAMM) was used to remove vocal characteristics that were not significant predictors of psychological distress. The choice of GAMM ensured that each predictor could be tested, allowing for nonlinearity with sex of caller as a moderating variable. The 2-level model reflected our approach to data collection: 40-ms frame voice characteristics (level 1) nested within individual calls (level 2), allowing for moderation by sex of caller.

Splines with differing degrees of freedom were added for each predictor to account for different forms of nonlinearity. Random intercepts were added to account for differences between individual callers. A binomial model with logit link was used to differentiate low from high psychological distress frames within calls.

### Clustering of Speech Frames Using Voice Characteristics

k-means clustering of speech frames was used to derive probabilistic clusters from the overall voice characteristics. This approach has been used when analyzing other forms of biologically derived data (eg, DNA gene expression) [20]. The addition of the principal components used to discriminate between clusters provided a supplementary predictor that improved classification accuracy in the next stage.

### Classification of Speech Frames Using Machine Learning

We sought to extend upon the work of Kandsberger [12] by using the reduced vocal characteristic predictor set (obtained via GAMM) to classify speech frames according to high and low psychological distress within the cluster configuration derived from the k-means clustering step.

Gradient boosting is a computationally inexpensive approach that is able to achieve a level of transparency unavailable to other powerful machine learning approaches (eg, support vector machines and neural networks). This transparency is desirable when clinical insights are of importance. In its base implementation, gradient boosting assumes linearity among predictors; however, this can be remedied with alternative implementations. Component-wise gradient boosting can analyze nonlinear data by first estimating a GAMM with smooth spline terms added and then by applying each model component (individual predictors and random components) to achieve the best reduction in classification error. Moderation by sex of caller was added to this model for all vocal variables.

Leave-one-caller-out cross-validation was used to validate the model, ensuring that speech frames from any single caller did not feature in both training and test sets. Ten-fold cross-validation was used to confirm the number of boosting trees, and the Youden J index was used to determine the optimal probability cutoff that maximized upon both precision and recall measures [21]. The efficacy of the classification algorithm was measured using AUCROC and the area under the precision-recall curve to ensure robust measures of accuracy in the presence of possible data-class imbalance.

Plain language definitions of the vocal characteristics included in the final model have been included in Multimedia Appendix 2.

### Misclassification

Following classification of speech frames using the component-wise gradient boosting algorithm, misclassifications were inspected at the annotated segment level rather than at the individual frame level. This was done by classifying each segment as high or low distress based on the mean probabilities for all the corresponding segment frames.

This approach ensured that the mental status examination descriptions made against each segment by the team of psychologists could be inspected to determine whether any patterns in caller presentation might be evident for the misclassified segments.

### Ethics Approval

No contact information for callers was obtained, and thus a waiver of consent was granted by the institutional human research ethics committee (reference #20214340-5805) in compliance with the Declaration of Helsinki. This study followed the STROBE (Strengthening the Reporting of Observational Studies in Epidemiology) reporting guidelines. A STROBE checklist is included in Multimedia Appendix 3.

## Results

### Overview

The sample comprised 87 female and 33 male callers (mean caller age 39.47 years, SD 15.24). In total, 117,387 40-ms frames (low psychological distress=75,504; high psychological distress=41,88; mean distress rating 4.92, SD 2.13) were nested within 1177 annotated call segments (low psychological distress=754, high psychological distress=423; mean number of segments per call 7.70, SD 4.22) and within 120 individual callers.

### Reduced Predictor Set via GAMM

**Figure 1:**
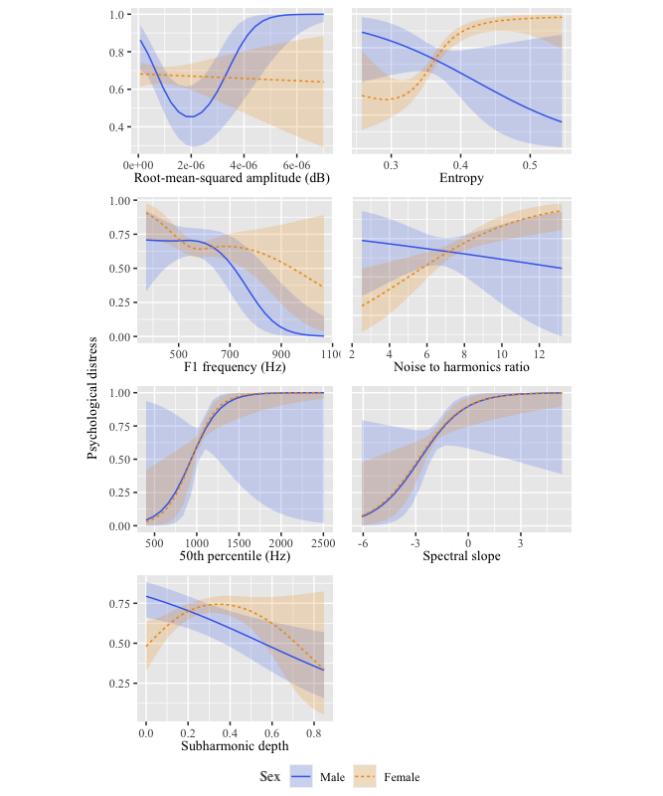
Relationship of each voice characteristic to the probability of high psychological distress.

Penalized GAMM was used to reduce and validate the number of candidate predictors while allowing for nonlinearity and accounting for moderation by sex of caller. The model overall accounted for 15.5% of the variance in the level of psychological distress (adjusted R^2^=0.15). Table 1 summarizes the coefficients and degree of nonlinearity of each of the 19 vocal characteristics included in the model, and the differences between male and female callers for each of the significant predictors is illustrated in Figure 1.

**Table 1:** Summary of voice characteristic coefficients in the generalized additive mixed-effects regression model^a^ (adjusted R2=0.15; n=117,387).

Variable			*β*		SE		95% CI		edf^b^		*F* test (*df*)		*P* value
**Male**													
	Root-mean-squared amplitude (dB)	8.40		0.08		8.24 to 8.56		2.30		6.21 (2,1455)		<.001	
	Dominant frequency (Hz)	2.62		0.05		2.54 to 2.71		1.00		2.25 (1,1455)		.13	
	Entropy	–2.40		0.05		–2.50 to –2.31		1.00		1.70 (1,1455)		.19	
	First formant frequency (Hz)	–6.05		0.05		–6.15 to –5.95		2.44		5.07 (2,1455)		.01	
	First formant width (Hz)	1.00		0.03		0.95 to 1.05		1.00		0.98 (1,1455)		.32	
	Second formant frequency (Hz)	–1.20		0.03		–1.26 to –1.14		1.00		1.14 (1,1455)		.29	
	Second formant width (Hz)	1.09		0.02		1.05 to 1.13		1.00		1.77 (1,1455)		.18	
	Third formant frequency (Hz)	–1.11		0.02		–1.16 to –1.07		1.00		1.63 (1,1455)		.20	
	Third formant width (Hz)	.77		0.04		0.69 to 0.84		1.90		1.91 (2,1455)		.11	
	Spectral flux	–1.19		0.05		–1.28 to –1.10		1.00		0.44 (1,1455)		.51	
	Noise to harmonics ratio	–0.58		0.04		–0.65 to –0.51		1.00		0.18 (1,1455)		.67	
	Loudness (sone)	.19		0.05		0.08 to 0.29		1.00		0.01 (1,1455)		.93	
	Spectral novelty	1.33		0.03		1.26 to 1.39		1.00		1.03 (1,1455)		.31	
	Peak frequency (Hz)	6.06		0.09		5.88 to 6.25		1.00		2.72 (1,1455)		.10	
	25th percentile frequency (Hz)	4.91		0.18		4.55 to 5.27		1.00		0.47 (1,1455)		.49	
	50th percentile frequency (Hz)	9.38		0.19		9.01 to 9.76		1.00		1.63 (1,1455)		.20	
	75th percentile frequency (Hz)	5.66		0.13		5.41 to 5.91		1.00		1.29 (1,1455)		.26	
	Roughness	1.59		0.03		1.54 to 1.65		1.00		2.24 (1,1455)		.14	
	Spectral centroid (Hz)	–22.60		0.51		–23.60 to –21.59		1.00		1.28 (1,1455)		.26	
	Spectral slope (Hz)	6.62		0.10		6.41 to 6.82		1.00		2.69 (1,1455)		.10	
	Depth of the subharmonics (Hz)	–1.56		0.01		–1.59 to –1.53		1.00		8.56 (1,1455)		.004	
**Female**													
	Root mean squared amplitude (dB)	–.17		0.02		–0.20 to –0.13		1.00		0.05 (1,1455)		.82	
	Dominant frequency (Hz)	1.79		0.04		1.71 to 1.87		1.00		1.39 (1,1455)		.24	
	Entropy	3.23		0.04		3.16 to 3.29		2.75		13.09 (3,1455)		<.001	
	First formant frequency (Hz)	–1.79		0.03		–1.86 to –1.73		2.87		1.98 (3,1455)		.08	
	First formant width (Hz)	–.72		0.02		–0.75 to –0.69		1.00		1.20 (1,1455)		.27	
	Second formant frequency (Hz)	.07		0.02		0.04 to 0.10		1.00		0.01 (1,1455)		.91	
	Second formant width (Hz)	3.04		0.04		2.97 to 3.12		2.67		2.28 (3,1455)		.07	
	Third formant frequency (Hz)	.03		0.02		0.00 to 0.07		1.00		0.03 (1,1455)		.96	
	Third formant width (Hz)	–0.83		0.02		–0.86 to –0.79		1.00		1.19 (1,1455)		.28	
	Spectral flux	2.45		0.04		2.38 to 2.52		1.00		3.13 (1,1455)		.08	
	Noise to harmonics ratio	2.45		0.02		2.41 to 2.49		1.00		9.49 (1,1455)		.002	
	Loudness (sone)	–.90		0.03		–0.97 to –0.84		1.00		0.46 (1,1455)		.50	
	Spectral novelty	.07		0.03		0.02 to 0.13		1.00		0.01 (1,1455)		.94	
	Peak frequency (Hz)	–2.09		0.05		–2.19 to –1.98		1.00		0.98 (1,1455)		.32	
	25th percentile frequency (Hz)	–3.48		0.11		–3.68 to –3.27		1.00		0.72 (1,1455)		.40	
	50th percentile frequency (Hz)	10.49		0.10		10.28 to 10.69		1.00		6.77 (1,1455)		.009	
	75th percentile frequency (Hz)	–4.37		0.08		–4.53 to –4.21		1.00		1.87 (1,1455)		.17	
	Roughness	–.11		0.02		–0.14 to –0.07		1.00		0.03 (1,1455)		.87	
	Spectral centroid (Hz)	–13.63		0.29		–14.20 to –13.06		1.00		1.45 (1,1455)		.23	
	Spectral slope (Hz)	6.53		0.06		6.40 to 6.65		1.00		6.91 (1,1455)		.009	
	Depth of the subharmonics (Hz)	–.87		0.03		–0.92 to –0.82		2.25		5.25 (2,1455)		.005	

^a^Male versus female: *β*=–.06; SE 0.25, 95% CI –0.44 to 0.56; *t*=–0.24; *P*=.81.

^b^edf: estimated degrees of freedom.

Although sex of caller was not a significant moderator for the model overall (*β*=–0.06; SE 0.25, 95% CI –0.44 to 0.56; *t*=–0.24; *P*=.81), the profile of significant vocal characteristics did differ between male and female callers. Male callers in our sample appeared to speak louder with increasing psychological distress (*β*=8.40, 95% CI 8.24 to 8.56; estimated degrees of freedom [edf]=2.3; *F_2.1455_*=6.21; *P*<.001). The frequencies of the first formant were also found to fall, suggesting an increase in the articulatory quality of vowel sounds (*β*=–6.05; 95% CI –6.15 to –5.95; edf=2.44; *F_2,1455_*=5.08; *P*=.01), while the depth of the subharmonics among male callers was also found to fall, suggesting increasing roughness of speech (*β*=–1.56, 95% CI –1.59 to –1.53; edf=1.00; *F_1,1455_*=8.56; *P*=.004).

In contrast, the voice quality of female callers was characterized by increasing entropy values, which are commonly associated with a decrease in vocal clarity (*β*=–3.23; 95% CI 3.26 to 3.29; edf=2.75; *F_3,1455_*=13.09; *P*<.001) and an increase in the noise to harmonics ratio, which indicates a greater proportion of noise components within the voice signal (*β*=–2.45; 95% CI 2.41 to 2.49; edf=1.00; *F_1,1455_*=9.49; *P*=.002). There was also an upward shift in the first half of frequencies amongst female callers (*β*=–10.49, 95% CI 10.28 to 10.69; edf=1.00; *F_1,1455_*=6.77; *P*=.009) in conjunction with an increase in spectral slope or breathiness of speech (*β*=–6.53; 95% CI 6.40 to 6.65; edf=1.00; *F_1,1455_*=6.91; *P*=.009). Similar to male callers, the depth of the subharmonics was also seen to fall, suggesting an increase in the roughness of speech (*β*=–.87, 95% CI –0.92 to –0.82; edf=2.25; *F_2,1455_*=5.25; *P*=.005).

### Clustering of Voice Characteristics

**Figure 2:**
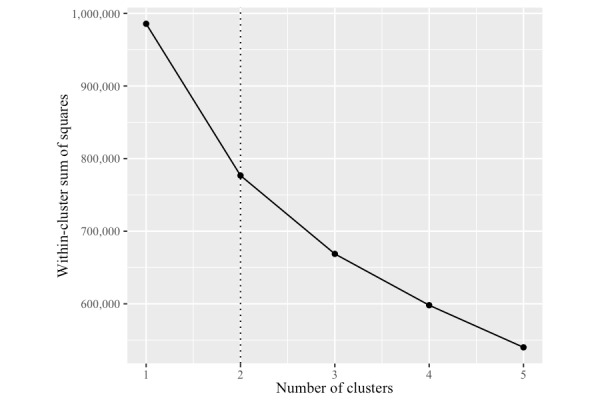
Scree plot of variance explained by different cluster configurations.

k-means clustering was used to reveal the probabilistic groupings that might be apparent within the voice characteristics data set. The reduced set of 7 vocal characteristics was used in a range of clustering configurations. Figure 2 illustrates a scree plot with different cluster configurations (clusters 1-5). The variance explained by the cluster configurations appears to level off after 2 clusters. The 2-cluster configuration was poorly associated with sex of caller (Cramer’s V=0.02), suggesting that both clusters were present within the vocal frames of male and female callers.

Binomial logistic regression (logit link) was used to validate the 2-cluster model. The full model accounted for 23.4% of the variance in the 2-cluster response variable. Model coefficients have been summarized in Multimedia Appendix 4. Speech frames in cluster 1 were characterized by higher values of entropy, first formant frequencies, 50th percentile frequency, and spectral slope values, while speech frames in cluster 2 were characterized by higher values in noise to harmonics ratio and the depth of the subharmonics. All call recordings had a mix of cluster 1 and 2 speech frames. The 2 principal component variables used to optimally separate the 2 clusters were added to the component-wise gradient boosting algorithm as additional predictors in the analysis. The subsequent classification model was first trialed without the cluster variables and achieved levels of classification accuracy of 75%, which increased to 94% when the cluster variables were included, validating the inclusion of this clustering step.

### Classification of Psychological Distress Speech Frames Using Component-Wise Gradient Boosting

Component-wise gradient boosting was used to classify each speech frame according to precoded high versus low psychological distress on the basis of the 2-cluster configuration and the 7 voice characteristics extracted. Leave-one-caller-out cross-validation results are reported below. The gradient boosting model classified each 40-ms speech segment to either high or low psychological distress categories with an AUROC=97.39% (95% CI 96.20-98.45) and an AUCPR=97.52 (95% CI 95.71-99.12). Thus, 39,282 of 41,883 (93.79%) speech frames nested within 728 of 754 (96.6%) segments were correctly classified as exhibiting low psychological distress. Conversely, 71,455 of 75,503 (94.64%) speech frames nested within 382 of 423 (90.3%) segments were correctly classified as exhibiting high psychological distress. In terms of variable importance, the random effect for individual callers contributed 68% to the variation in level of psychological distress in the classification algorithm, followed by cluster configuration (23.4%), spectral slope (4.4%), and the 50th percentile frequency (4.2%)

### Misclassification

After accounting for moderation effects by sex of caller, our component-wise gradient boosting classification algorithm incorrectly classified 2601 of 41,883 (6.21%) frames nested within 26 of 754 (3.5%) segments as exhibiting low psychological distress, while 4049 of 75,504 (5.36%) frames nested within 41 of 423 (9.7%) segments were misclassified as exhibiting high psychological distress. A review of the mental status examination descriptions that were made by the reviewing team of psychologists suggested that these call segments were often typified by considerable emotion with high anxiety but were voiced in whispered or soft tones. These particular presentations might have masked the vocal characteristics used to successfully classify the majority of the other recordings.

## Discussion

Voice characteristics coupled with artificial intelligence has the potential to yield highly accurate models for the classification of emotional states. Our aim in this novel study was to classify 40-ms speech frames sourced from helpline counseling telephone calls according to high versus low levels of psychological distress. By achieving this aim to a high level of accuracy, we demonstrate an efficient, economical, and scalable approach to the detection of psychological distress in an ecologically valid telehealth setting.

We developed an ensemble approach to achieve an optimal outcome. This approach identified and validated a range of candidate vocal characteristics via penalized GAMM, which together elucidated a set of 7 characteristics (from the initial 19) with a strong predictive relationship to the binary outcome measure (clinical levels of psychological distress) when allowing for sex as a moderator. This analysis also yielded a number of heuristics that illustrate how each voice characteristic changes in response to a shift from high to low psychological distress, providing important clinical insights. In the second phase of the analysis, k-means clustering combined with component-wise gradient boosting succeeded in accurately classifying the 40-ms speech frames (AUROC=97.39%). Although misclassifications did occur, these were largely confined to a minority of annotated segments within calls (67 of 1177 segments, 5.79%) that often featured anxiety or whispered tones. This suggests that specific caller presentations may not translate as well to vocal-informed classifications of distress, particularly when the level of distress is masked in the caller’s voice.

We have achieved comparable results to Kandsberger and colleagues [12] who found significant differences (*P*<.05) between high and low psychological distress when the fundamental frequency of participants’ speech was measured. Although we were unable to analyze the fundamental frequency of callers (unavailable within the frequency range of telephone calls), we did obtain significant effects for male and female callers separately on other voice characteristics, including root-mean-squared amplitude, first formant frequencies, and the depth of the harmonics among male callers; and entropy, noise to harmonics ratio, the 50th percentile of frequencies, spectral slope, and the depth of the subharmonics among female callers.

Specifically, with a shift from high to low psychological distress, male callers appeared to speak with increasing average loudness, greater vowel articulatory quality (lower first formant frequencies), and with greater roughness of speech (lower subharmonic depth). In contrast, the voice of female callers was characterized by a decrease in vocal clarity (entropy), an increase in noise in the vocal signal (noise to harmonics ratio), higher frequencies within the first half of the frequency spectrum, increasing breathiness of speech (spectral slope), and increasing roughness of speech (subharmonic depth).

However, we differed substantially from the approaches of previous authors in a number of important ways. First, we measured psychological distress using multiple sources of ratings rather than inferring it via the presence of psychopathology as previous authors have done; second, we employed an ensemble approach to ensure an optimal set of voice characteristics, allowing for nonlinear relationships; third, our use of k-means clustering has revealed probabilistic groupings within each call recording, and finally we have trained and tested a machine learning algorithm that, in conjunction with the 7 critical voice characteristics, has resulted in a more accurate classification of psychological distress. Furthermore, we have demonstrated our approach in an ecologically valid setting of telehealth, replete with background noise and variable call quality.

However, our investigation is not without limitations. We assessed the level of psychological distress using clinical researcher ratings. However, we were unable to ascertain the true level of distress experienced first-hand by the callers themselves due to the practical and ethical issues raised in terms of querying the emotional experience of callers directly when other more pressing issues were at hand (eg, imminent risk of suicide). Instead, we have relied upon objective assessments of psychological distress by trained personnel, which may indeed have advantages over a subjective assessment by callers.

We were able to measure and analyze the quality of caller’s voice in terms of frequency across formants and in terms of loudness, breathiness, and clarity. However, we were not able to assess other dimensions of caller’s speech, such as timing of speech. This might be important given that other authors have noted a slower speaking style in both depressed and suicidal cohorts [14]. Finally, it might be possible to achieve higher levels of accuracy with other powerful machine learning approaches, such as support vector machines and neural networks. However, this would also sacrifice the level of the transparency we have achieved with our hybrid approach.

Our investigation also has a number of strengths. Similar to Kandsberger and colleagues [12] we analyzed a sample of recordings obtained from ecologically valid settings. In this way, previous research findings, such as those obtained by Scherer and colleagues [11], have been taken out of clinical settings and trialed within more naturalistic helpline conditions that are often typified by poor call quality and the presence of background noise. We have also classified short 40-ms frames of speech, rather than individual calls at the holistic level, according to high versus low psychological distress. That we have done so to a high level of accuracy suggests the possibility of real-time detection of psychological distress among helpline callers. This is important if such technology is to realize its full potential as a clinical decision aid with the potential for early intervention, such as call triaging. In addition, it provides a method for the measurement of changes in distress over the duration of helpline calls which could be used to evaluate helpline outcomes.

## Appendix

Multimedia Appendix 1 Analysis flowchart.

Multimedia Appendix 2 Plain language definitions of final model vocal characteristics.

Multimedia Appendix 3 STROBE (Strengthening the Reporting of Observational Studies in Epidemiology) checklist.

Multimedia Appendix 4 Validation of cluster configuration via binomial logistic regression.

## Abbreviations

AUROC: area under the receiver operating characteristic
DSM: Diagnostic and Statistical Manual of Mental Disorders
edf: estimated degrees of freedom
GAMM: generalized additive mixed-effects regression model
PTSD: posttraumatic stress disorder
STROBE: Strengthening the Reporting of Observational Studies in Epidemiology

## Data Availability

The data sets generated during or analyzed during the current study are not publicly available due the private and confidential nature of the counseling calls investigated and the possibility of reidentification of the vocal based upon the vocal characteristics reported.
