# Supplementary figures and images for: Using Vocal Characteristics To Classify Psychological Distress in Adult Helpline Callers: Retrospective Observational Study
Source: JMIR Form Res. 2022 Dec 19;6(12):e42249. doi: 10.2196/42249 (PMC9811648; doi:10.2196/42249)

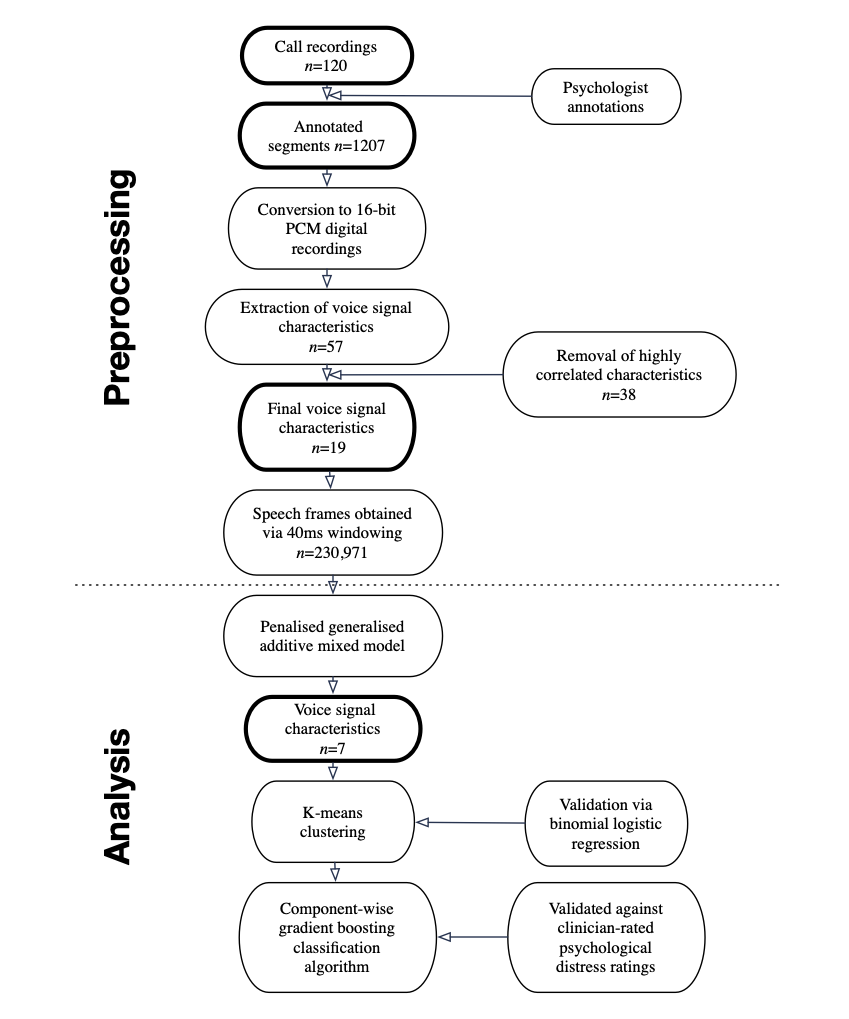

Supplement: Multimedia Appendix 1 [file formative_v6i12e42249_app1.png]
