# Supplementary material for: Using Vocal Characteristics To Classify Psychological Distress in Adult Helpline Callers: Retrospective Observational Study
Source: JMIR Form Res. 2022 Dec 19;6(12):e42249. doi: 10.2196/42249 (PMC9811648; doi:10.2196/42249)
Supplement: Multimedia Appendix 2 [file formative_v6i12e42249_app2.pdf]

Variable Name	Description
Root Mean Squared Amplitude (dB)	Root mean squared Amplitude. The mathematical operations – Square root, mean, then squared, ensure that both the negative and positive amplitude segments (either side of zero amplitude on a normal sound wave signal) are measured. Thus, it reflects the 'absolute' amplitude or loudness.
Entropy (spectral)	Measured on a scale of 0 – 1, where values closer to =0 suggest a clarity of voiced signal, while values closer to =1 suggest silence or white noise..
Formant Frequency (Hz)	Vocal formants are concentrations of acoustic energy around a particular frequency with one formant occupying approximately every 1000Hz (e.g. first formant = 0-1000hz). The height of the first formant has been associated with vowel articulation, such that lower first formant values are associated with higher vowel height (i.e. vowels that require the tongue to be positioned closer to the roof of the mouth, thus requiring greater quality of articulation).
Noise to harmonics Ratio (dB)	The Noise to harmonics Ratio is used to differentiate harmonic from noise components of speech and is expressed in dB. Lower values indicate a greater proportion of noise like components that might originate from unstable vocal fold vibration which may interfere with the normal rhythmicity of vibration. Higher values indicate a greater proportion of the signal being within the harmonics.
Modulation spectrum	The modulation spectrum is a translation of the vocal to a 2-dimensional representation of time (x-axis) versus frequency (y-axis)
50th Percentile Frequency (Hz)	The frequencies corresponding with the 50th Percentile (median) of the modulation spectrum (see above). Higher values indicate a shift in median frequencies to higher pitch registers.
Spectral Slope	The slope of the linear regression line of best fit of the frequencies in the modulation spectrum (see above). Increasing slope coefficients signify increasing physiological effort expended to create voice. This increase in physiological effort is associated with a more breathy vocal quality.
Depth of Subharmonics (Hz)	Estimated depth of the subharmonics in Hz. The presence of subharmonics i.e. lower values have been associated with turbulent vocal fold vibrations and with the perception of roughness of voiced speech.
