# Supplementary material for: Using Vocal Characteristics To Classify Psychological Distress in Adult Helpline Callers: Retrospective Observational Study
Source: JMIR Form Res. 2022 Dec 19;6(12):e42249. doi: 10.2196/42249 (PMC9811648; doi:10.2196/42249)
Supplement: Multimedia Appendix 4 [file formative_v6i12e42249_app4.pdf]

| Variables | $\beta$ | *se* | Odds Ratio | 95% CI | Z-value | P-value |
|---|---|---|---|---|---|---|
| Root Mean Squared Amplitude (Hz) | 0.00 | 0.01 | 1.00 | (0.99, 1.01) | 0.31 | 0.76 |
| Entropy | 0.05 | 0.02 | 1.06 | (1.01, 1.10) | 2.61 | <.01 |
| First Formant Frequency (Hz) | 0.21 | 0.01 | 1.23 | (1.20, 1.26) | 17.11 | <.001 |
| Noise to harmonics ratio | -0.25 | 0.01 | 0.78 | (0.76, 0.80) | -20.70 | <.001 |
| $50^{th}$ Percentile Frequency (Hz) | 0.58 | 0.01 | 1.79 | (1.75, 1.83) | 52.45 | <.001 |
| Spectral Slope (Hz) | 0.02 | 0.01 | 1.02 | (1.00, 1.03) | 2.49 | <.05 |
| Subharmonics depth (Hz) | -0.04 | 0.01 | 0.96 | (0.95, 0.97) | -7.04 | <.001 |
